# Structuring targeted surveillance for monitoring disease emergence by mapping observational data onto ecological process

**DOI:** 10.1098/rsif.2013.0418

**Published:** 2013-09-06

**Authors:** Luca Gerardo-Giorda, Gavino Puggioni, Robert J. Rudd, Lance A. Waller, Leslie A. Real

**Affiliations:** 1 Center for Disease Ecology, Rollins School of Public Health, Emory University, Atlanta, GA, USA; 2 Department of Biology, Rollins School of Public Health, Emory University, Atlanta, GA, USA; 3 Department of Biostatistics and Bioinformatics, Rollins School of Public Health, Emory University, Atlanta, GA, USA; 4 Rabies Lab, Wadsworth Laboratories, New York State Health Department, Albany, NY, USA

**Keywords:** public health, risk monitoring, raccoon rabies, modelling, data assimilation

## Abstract

An efficient surveillance system is a crucial factor in identifying, monitoring and tackling outbreaks of infectious diseases. Scarcity of data and limited amounts of economic resources require a targeted effort from public health authorities. In this paper, we propose a mathematical method to identify areas where surveillance is critical and low reporting rates might leave epidemics undetected. Our approach combines the use of reference-based susceptible–exposed–infectious models and observed reporting data; We propose two different specifications, for constant and time-varying surveillance, respectively. Our case study is centred around the spread of the raccoon rabies epidemic in the state of New York, using data collected between 1990 and 2007. Both methods offer a feasible solution to analyse and identify areas of intervention.

## 1. Introduction

As pointed out in *Microbial threats to health: emergence, detection and response* [1], the degree of success of global and national efforts to create public health infrastructure with effective systems of surveillance and response is a key variable influencing the future impact of infectious diseases. According to WHO, surveillance is *an ongoing, systematic collection, analysis and interpretation of health-related data essential to planning, implementation and evaluation of public health practice* (http://www.who.int/immunization_monitoring/burden/routine_surveillance/en/index.html). Surveillance plays a major role in devising public health strategies to curtail the spread of infectious diseases and early detection remains the first line of defence in preventing the emergence of novel disease outbreaks. Often, surveillance is the decisive factor in triggering early intervention [2,3], in order to avoid the higher public health costs associated with a widespread infection in the case an outbreak has gone undetected.

The definition of an epidemic/epizootic or outbreak is varied and has a long history of confusion (see Rosenburg [4] for an account of the history of the concept of an epidemic). Contemporary discussions have assumed at least two definitions of epidemic or outbreak occurrence. Childs *et al.* [5], for example, consider a rabies outbreak as occurring when the observed number of cases falls above a baseline for a specified number of consecutive observation periods and where the average number of cases in a given location determines the base line. They suggest an above-average reported rate at the county level for three consecutive months. The other most common definition treats any occurrence of an infectious disease as an outbreak, where it is detected in a novel geographical location and poses a significant public health threat, because of its novel appearance in that location. Throughout this paper, we adhere to this latter definition since we are concerned with uncovering appropriate surveillance strategies for detecting novel occurrences of disease.

Resources for infectious disease surveillance are always in limited supply and any strategy that provides insights into the optimal guidance of surveillance programmes is a valued addition to our public health infrastructure [6]. Guidance strategies should include the identification of both areas and populations that are at increased risk of disease exposure. This is the key idea associated with the concept of *targeted surveillance* (also known as *risk-based surveillance*) defined as a surveillance strategy that focuses sampling on high-risk populations in which specific and commonly known risk factors exist [7]. The concept of targeted surveillance was first formally introduced following the emergence of bovine spongiform encephalopathy (BSE) in the UK during the 1996 epidemic [8]. This idea is also behind the recently emerging field of model-guided surveillance [9].

In the USA, the Council of State and Territorial Epidemiologists, in collaboration with the Center for Disease Control (CDC), maintains a list of notifiable diseases constituting the National Notifiable Diseases Surveillance System. For human diseases, healthcare providers are an essential component of any surveillance programme, but their impact is significantly reduced when confronted with an epidemic of zoonotic origin. Monitoring of wildlife reservoirs is an essential component of detection but rarely undertaken routinely. What we understand of zoonotic epidemics is largely constructed from passive reporting of occurrences gleaned from haphazard and incomplete surveillance of animal populations usually as the result of an animal–human interaction [10]. For the purposes of our analysis, the reporting rate (or equivalently the detection rate) is taken to constitute the fraction of reported cases over the total number of infections. Reporting rates vary significantly over both time and space and may deviate significantly from the true underlying distribution of infections due to a variety of sources (e.g. variation in the size and extent of infection clusters, heterogeneity in human and host population densities, etc. [11]). However, these factors explain only partially the spatial and temporal heterogeneity in reporting rate. Variation in the implementation and structure of surveillance programmes can themselves be a significant source of reporting rate variation and a mapping of different levels of reporting rate and surveillance efforts across space or time can help identify specific areas in need of intervention.

A variety of mathematical models are available in the literature to describe the dynamics of infectious diseases using the generalized susceptible–exposed–infectious–removed (SEIR) modelling structure (see, for instance, [12,13] or, for a spatially continuous model, [14]), and some work has been done at estimating the reporting rates for some human diseases confering lifelong immunity [15,16], but little effort has been directed at elucidating how to incorporate reporting data into models of surveillance [17], especially from an ecological viewpoint [18]. The goal of this paper is to show how to use reporting data (both reports of positive and negative occurrences) to identify geographical areas where surveillance levels are potentially insufficient to detect outbreaks.

Our approach is intended to provide a useful tool for public health agents, who monitor critical areas for surveillance and allocate funds for increased intervention. We introduce two different methods depending on whether agents have fixed or time-varying reporting rate data. The first method is based on a simple, constant reporting rate, intended to model a constant level of surveillance over time. Considering that surveillance levels usually change as a consequence of case detection and local public health concerns, we relax this assumption in our second method, where we formulate a reporting rate that changes over time and depends on the total number of reports (positive and negative) and the estimated host population. Provided that such an estimate is moderately accurate at any given time, it is possible to track disease dynamics through a model for infectious spread. The first approach identifies a surveillance risk, whereas the second one identifies a surveillance efficacy. The concepts are not mutually exclusive, and the observed correlation between our results from the two approaches supports their mutual consistency. As a consequence, either method can be used to identify areas where surveillance levels are critical, possibly underassessed and potentially leaving an outbreak unidentified. Such evaluation relies on comparing the values of computable parameters (risk or efficacy) across different counties. From the public health standpoint, the areas identified by the method as *at risk* are the ones where additional resources should be allocated for targeted monitoring. The proposed models provide input for explicit assessment of which counties need active intervention by public health decision-makers.

The approach we introduce combines process-driven and observational methods. It is quite general and suitable to a wide range of infectious disease systems and datasets. Moreover, it has great potential for application to human diseases. The approach relies on good estimates of the population size and a good knowledge of the epidemiology of the disease. Both aspects are crucial, and often poorly specified. In the case of human diseases, the knowledge of the susceptible population and mitigated uncertainty about the epidemiological parameters of the disease would significantly increase the accuracy of the method. The model then can serve as a basis to improve surveillance strategies, particularly in disadvantaged regions.

For illustrative purposes, we apply our method specifically to the spread of raccoon rabies virus among its raccoon (*Procyon lotor*) hosts in the state of New York. Rabies, a viral encephalomyelitis specific to mammals, has been a CDC notifiable disease since the mid-1970s. Rabies has the longest extant record of reports of any zoonotic disease in the USA. Rabies virus is transmitted from one animal to another usually by a bite [19,20]. Because its transmission modality is favourable to interspecies infection, including human beings, rabies is a major public health concern. Raccoons are the major terrestrial vector of the disease in the eastern USA, though many foxes, bats and skunks carry the disease as well [10,21]. The potential risks to humans coupled with an extensive database with high geographical resolution, exact occurrence dates and knowledge of the species of host involved engenders the application particularly relevant and amenable to testing our methods and approach.

## 2. Material and methods

### 2.1. Model

We consider the dynamics of a lethal disease, as described by a compartmentalized model of susceptible–exposed–infectious (SEI) type. The model subdivides the population into susceptible, exposed (hosts that have been exposed to the virus but not yet infectious) and infectious (host with the capability of transmitting the pathogen). The spatial resolution of the model is set at regional level (from township to state). Consequently, the computational model consists of a system of ODEs
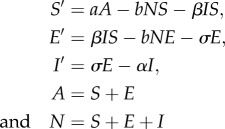
completed by suitable initial conditions. In the above equations, we denote by *β* the transmission of pathogen by contact between a susceptible and an infectious individual, by *v* the vaccination rate, by *σ* the reciprocal of the latency period, by *α* the reciprocal of the life expectancy of an infectious host.

We assume a density-dependent mortality rate in the absence of the disease, *bN*. We denote by *a* the reproduction rate, which represents a yearly average, to take into account the reduced fecundity of juveniles. Seasonality is not explicitly included here, but could easily be with a time-dependent reproduction rate [22]. Moreover, we assume that only susceptible and exposed individuals are able to reproduce. Such an assumption is reasonable for a very aggressive disease in wildlife, assuming the expected survival of an infectious host much too short to give birth or care of the offspring. To show the dynamics of the epidemic model, we ran a simulation of the SEI model within one single virtual region. We report in table 1 the model parameter values that are adapted to raccoon rabies for the eastern USA and have either been drawn from published values and US Department of Agriculture sources (http://www.usda.org) or estimated indirectly. In particular, the birth rate *a*, the transmission rate *β*, the latency period 1/*σ* and the infectious period 1/*α* are taken from literature [25–29]. The rate of density-dependent mortality, *b*, is estimated indirectly to produce a disease-free equilibrium of 27 000 individuals, corresponding to a density of 11 animals per km^2^ (average for raccoons in the eastern USA [23]) in a region of 2457 km^2^ (average size of a New York county, outside the five boroughs of New York city). We simulate 922 weeks of epizootic. The plot of the temporal dynamics of the full SEI model (susceptible, exposed, infectious and total population) is available in the electronic supplementary material.

**Table 1.** Coefficients of the SEI model. The natural death rate is chosen to be density dependent to provide a carrying capacity compatible with the published values in literature of 5–17 animals per km^2^ [23,24]. The birth rate *a*, the transmission rate *β*, the latency period 1/*σ* and the infectious period 1/*α* are taken from [25–29].

*a*	birth rate	2.67 k/f/y
*μ*_0_	natural death rate	variable
*β*	contact rate	1×10^–4^ (a d)^–1^
1/*σ*	latency period	50 days
1/*α*	infectious period	14 days

In order to simplify the dynamics of the SEI system, we aggregate the model to a planar system in terms of the infectious individuals *I* and the total population *N*. Since *A* = *N* − *I*, by summing up the first three equations in the model, we get
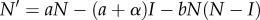
and
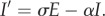


A fourth class of removed could be included in a more general model, consisting of hosts that recovered from the disease or have been vaccinated. Since there is no evidence for natural recovery in rabies, which is our case study in this paper, and we do not consider vaccination at this level, the removed class is not considered. However, the following results are based on an aggregated method, and the use of a SEIR model would not affect the conclusions.

### 2.2. Main features of the aggregated model

The aggregated model is not in closed form due to the presence in the second equation of the term *σE*. However, the knowledge of the new infectious *σE* temporal dynamics is sufficient to reproduce the dynamics of the full SEI model by means of the aggregated one. If the new infectious are known as function of time, their dynamics can be considered a source term *Φ* in the second equation of the aggregated model, which can be written in the more general form
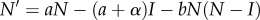
and
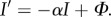


**Figure 1.**
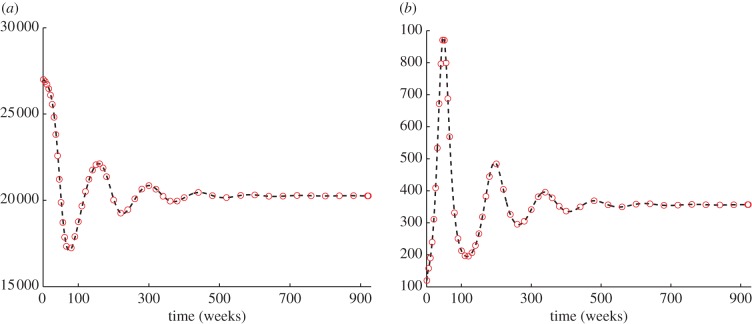
Comparison between the temporal dynamics of (*a*) total population and (*b*) the infectious for the complete SEIR (dashed line) and the aggregated model (*N*, *I*) (bullets).

To support our claim, we ran a simulation of the reduced model using as a source term in the second equation the temporal dynamics of the new infectious *σE*, obtained by simulating the full SEI. We compare in figure 1 its dynamics with those of the aggregated model. We plot the dynamics of both the total population (figure 1*a*) and the number infectious (figure 1*b*). In both pictures, the dashed line represents the values obtained with the full SEI model, whereas the circles represent the values obtained with the aggregated model. The numerical results confirm that the knowledge of the temporal dynamics of the new infectious *σE* is sufficient to reproduce the SEI dynamics with the aggregated model.

**Figure 2.**
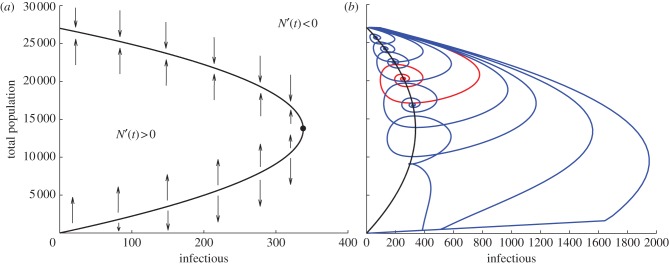
(*a*) *N*-nullcline and stability for constant values of infectious: the upper branch of the curve is stable, whereas the lower one is unstable. (*b*) Trajectories in the phase plane (*N*,*I*) associated with different temporal dynamics of the new infectious *Φ* = *ζ* × (*σE*), with *ζ* = 0.25, 0.5, 0.75, 1, 1.25, 1.5, 1.75, 2, and *σE* from the complete SEI model. In red, we highlight the trajectory asociated with *ζ* = 1, corresponding to the one of the complete SEI model.

A direct stability analysis for the aggregate model is not feasible. However, we can identify the *N*-nullcline, namely the set of points in the plane (*N*,*I*), where *N′* = 0, that is shown in figure 2*a*. If the number of infectious is constant, the upper branch of the nullcline is stable, whereas the lower branch is unstable. Moreover, as expected, the persistence of infectious hosts (i.e. an endemic state) reduces the carrying capacity of the host.

Different temporal dynamics of the new infectious *Φ* entail complex behaviours of the system in terms of epidemic outbreak, including persistency and possible extinction of the host population. We simulated different temporal dynamics by rescaling the new infectious from the full SEI, as *Φ* = *ζ* × (*σE*), with *ζ* = 0.25, 0.5, 0.75, 1, 1.25, 1.5, 1.75, 2. The resulting trajectories in the phase plane (*N*,*I*) are plotted in figure 2*b*. If the growth rate of the newly infectious hosts *Φ* is too large, the population goes extinct along the bisector of the phase plane *N* = *I* (note the different scales on the axes). Otherwise, the trajectories show different levels of population drops in epidemic outbreaks, and a recovery process towards the stable endemic equilibrium on the upper branch of the *N*-nullcline.

### 2.3. Modelling detection rate for surveillance

Effective surveillance within a region amounts to the ability to identify newly infectious individuals. In the SEI model, this amounts to the correct assessment of *σE*, and to estimate the surveillance levels in the different counties, we need an accurate evaluation of this value. However, this value is unknown. We propose to extrapolate the value *σE* from the available data in a given observational window, whose length we denote by *τ*. Specifically, we consider the reported positive and negative cases. We denote by **r**_+_(*t*) and **r***_−_*(*t*) the reported positive and negative cases at time *t*, respectively, and the total amount of reports (positive and negative) along the observation window *I_t_* = [*t* *− *τ**, *t*] are given by
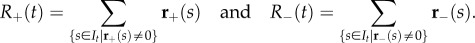
Note that the istantaneous reports **r**_+_(*t*) and **r***_−_*(*t*) are 0 for most times *t*, according to the reporting frequency of the public health departments. In what follows, the dependency on time will be left out.

We introduce a suitable function of the available reports that we denote with *F*(*R*_+_, *R_−_*), whose role is to expand the actual number of reported cases to take into account the effectiveness of the surveillance procedure, leading to the extrapolation model
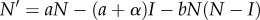
and
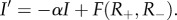


### 2.4. Compatibility of the extrapolation functions

The extrapolation function *F*(*R*_+_, *R_−_*) has to satisfy any compatibility requirements arising from the disease dynamics under consideration. Our case study in this paper concerns raccoon rabies, which is a lethal disease for the host, killing an infected animal within two weeks from the emergence of symptoms. For a lethal disease, the total population drop (namely the percentage of animals killed by the first outbreak) is known to be related to the basic reproductive rate *R*_0_ associated with the disease [30], and can be used as a compatibility constraint. We would like to observe that estimating the population drop with this method is not needed for most human diseases, as public health data regarding the number of deaths are usually available.

For the SEI model introduced earlier, the basic reproductive rate is given by
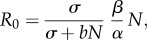
while the expected population drop [30] is
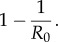
The reported values in literature for raccoon rabies *R*_0_ lie between 1.2 and 1.4 [26]. As a consequence, a population drop between 16% and 28% can be used as a reliable compatibility constraint for the system
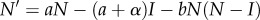
and
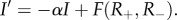


### 2.5. Modelling extrapolation

We propose here two different extrapolation functions to model surveillance efficacy that depend upon a family of parameters. The first models a constant level of surveillance, whereas the second models dynamic surveillance over time. We base our analysis on the assumption that an outbreak actually occurred in every area featuring positive reports.

#### 2.5.1. Constant surveillance

*Constant surveillance* in time is modelled using only the positive reports *R*_+_, together with a linear extrapolation function
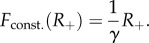
In the above expression, *γ* is the reporting rate, namely the percentage of new rabid cases that are actually detected.

Reporting activity varies in space and is also known to be correlated with the population density [10]. In order to identify the local surveillance efficacy for a given area, we express *γ* in terms of the human population density of the area (*h*)
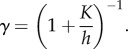
This choice models an increase in the reporting rate with the human density: in particular, if *h* is zero then *γ* vanishes, and as *h* increases *γ* approaches 1 (that is, in the case where human population density is infinite, every new infectious case would be detected). The positive parameter *K* is a risk index: the larger its value, the lower the reporting rate for a given human population density.

Knowing the initial population in a given area, we can identify the parameter *γ* fulfilling the compatibility requirements on the extrapolation function. In order to assess the level of surveillance in the region, we choose the corresponding risk index *K*.

We iterate the procedure over all the areas of interest and identify the corresponding values for *γ*. This procedure clearly depends on the epidemic under study. To eliminate such dependence, we normalize the risk index to a scale from 1 to 10, where a small value indicates a high level of surveillance in the region, whereas a large value entails a significant risk of an outbreak to go undetected in the area.

#### 2.5.2. Dynamic surveillance

*Dynamic surveillance* in time is modelled by using both positive *R*_+_ and negative *R_−_* reports, combined through a nonlinear extrapolation function
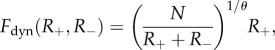
where *θ* > 1 is a parameter that represents the surveillance efficacy. The choice of the function *F*_dyn_(*R*_+_, *R_−_*) relies on two assumptions. First, we want a change in a small number of total reports to be more significant than a change in a larger number (a concept similar to diminishing returns in economics). Then, we assume that the testing procedure has sensitivity 1 (that is, if we could test all individuals we would be able to identify all the new infectious cases) and specificity 1 (we have no false positives). As a consequence, the function depends also on the total population *N*.

Also in this case, knowing the initial population, we can identify the parameter *θ* fulfilling the compatibility requirements on the extrapolation function. We iterate the procedure over all the areas of interest and identify the corresponding values for *θ*. In this case, a large value of *θ* indicates a high level of surveillance in the area, whereas a small value of *θ* highlights a significant risk that an outbreak can go undetected in the region.

### 2.6. New York State epidemiological data (1990–2007)

On 4 May 1990, the first case of a rabid raccoon was recorded in the state of New York, in Addison Township, Steuben County, on the New York/Pennsylvania border, as part of an advancing wavefront of rabies spread. By the end of 1994, the epizootic had propagated extensively across the state. The epizootic wave across NY was actually part of a larger epizootic that began at the boundary between Virginia and West Virginia in the mid-1970s and spread northeast through Pennsylvania and Connecticut and southeast to North Carolina [5], but entering NY in 1990.

At the time of the outbreak, rabies posed a particularly pressing public health problem with the number of post-exposure prophylactic treatments increasing from around 70 before the outbreak to over 1200 by 1991 [31]. Consequently, intensive surveillance and monitoring of wildlife populations was undertaken by the state and continues today. An extensive database has been collected by the New York State Department of Health. Each entry was recorded at the township level (754 locations) from 1990 to the present. The data we use in our analysis are those positive and negative cases verified by the New York State Department of Health from 1990 to 2007.

**Figure 3.**
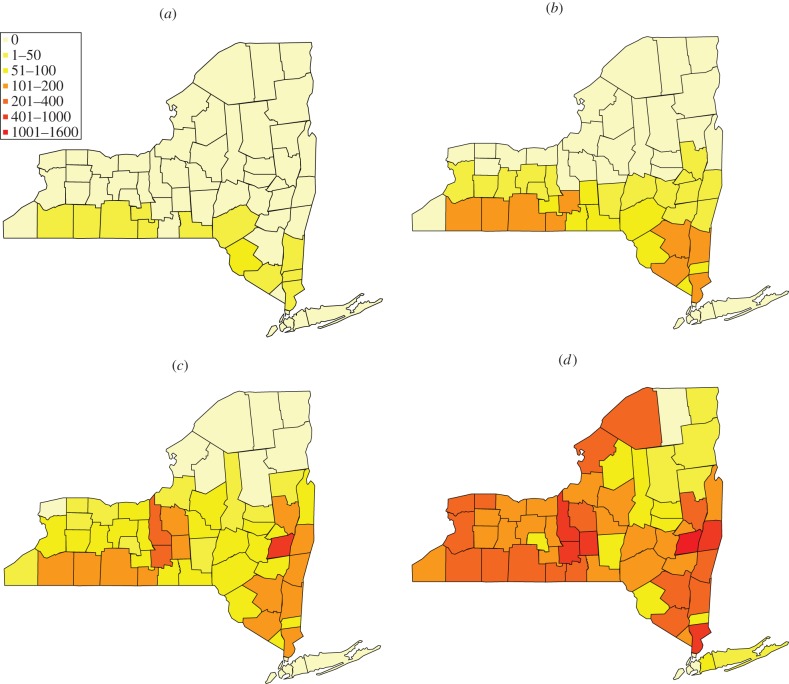
Total reported cases aggregated by county at different times. (*a*) 6 May 1991, (*b*) 30 November 1992, (*c*) 21 June 1994 and (*d*) 31 December 2007.

We aggregated the data at the county level, at which surveillance and intervention policies are actually implemented. Table 2 collects the 56 counties that featured reported cases of rabid raccoons in the period 1990–2007, their human population density and the total positive cases. Figure 3 illustrates the progression of the epidemic across the state at four different times, in terms of total reported cases at the county level.

**Table 2.** New York State epidemiological data (1990–2007). Counties, area, human population densities and total reported rabid cases from 1990 to 2007.

county	area (km^2^)	density	reports	county	area (km^2^)	density	reports
1. Albany	1380	213.45	1547	29. Oneida	3142	74.94	198
2. Allegany	2678	18.64	223	30. Onondaga	2088	219.51	290
3. Broome	1852	108.28	102	31. Ontario	1715	58.44	135
4. Cattaraugus	3393	24.74	237	32. Orange	2173	157.09	236
5. Cayuga	1797	45.61	688	33. Orleans	1013	43.6	208
6. Chautauqua	2751	49.59	191	34. Oswego	2468	49.59	171
7. Chemung	1064	85.59	230	35. Otsego	2598	23.74	114
8. Chenango	2328	22.08	85	36. Putnam	637	150.31	93
9. Clinton	2896	27.59	5	37. Rensselaer	1722	88.58	448
10. Columbia	1678	37.6	306	38. Rockland	515	556.8	120
11. Cortland	1300	37.38	403	39. St Lawrence	7306	15.32	223
12. Delaware	3802	12.64	132	40. Saratoga	2186	91.78	337
13. Dutchess	2137	138.11	265	41. Schenectady	544	269.4	145
14. Erie	2704	351.43	341	42. Schoharie	1621	19.48	160
15. Essex	4962	7.83	40	43. Schuyler	886	21.7	112
16. Fulton	1380	39.91	52	44. Seneca	842	39.6	120
17. Genesee	1282	47.09	117	45. Steuben	3636	27.15	222
18. Greene	1704	28.28	130	46. Suffolk	2362	600.92	17
19. Hamilton	4683	1.15	2	47. Sullivan	2582	28.65	98
20. Herkimer	3776	17.06	87	48. Tioga	1355	38.22	232
21. Jefferson	3294	33.92	223	49. Tompkins	1233	78.27	415
22. Lewis	3341	8.06	99	50. Ulster	3007	59.11	273
23. Livingston	1658	38.8	169	51. Warren	2253	28.1	34
24. Madison	1715	40.49	119	52. Washington	2191	27.86	198
25. Monroe	1707	430.78	124	53. Wayne	1564	59.95	465
26. Montgomery	1062	46.81	95	54. Westchester	1295	713.1	164
27. Nassau	743	1796.16	67	55. Wyoming	1544	28.12	121
28. Niagara	1355	162.25	285	56. Yates	974	25.28	86

### 2.7. Estimate of the raccoon population

One of the major limitations in studying wildlife epidemics is the difficulty in establishing the actual size of the at-risk population under investigation. Best estimates from the literature suggest that raccoon density in the eastern USA falls in the range of 5–17 animals per km^2^ [23,24].

**Figure 4.**
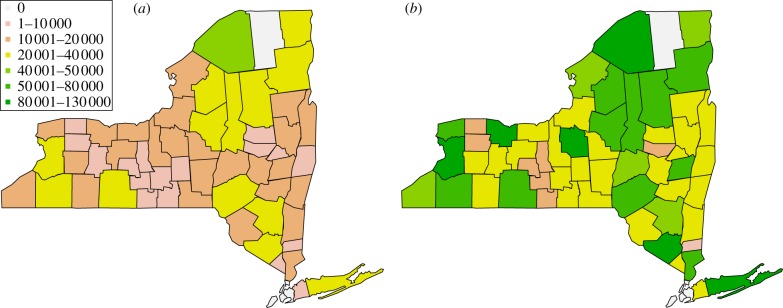
(*a*) Minimal and (*b*) maximal initial population stochastically generated in the 56 counties included in the study.

We consider in this study all 56 counties (table 2) that featured reported cases of rabid raccoons in the period 1990–2007. We mitigate the uncertainty about the actual raccoon population size by drawing, for each county, 50 values from a normal distribution with mean 11 and s.d. 2 (in order to cover the variability among the different ranges in the literature, see [23,24] and references therein). We add a correction to this distribution by taking into account the human population density: according to the New York State Department of Environmental Conservation (http://www.dec.ny.gov/animals/9358.html), raccoons are more prone to establish in areas where the human presence is higher. Suburban/metropolitan areas are often associated with the highest recorded raccoon population densities. We thus added an extra term to the counties with human density above the average for the state (157.81 individuals per km^2^), by adding draws from a normal distribution with mean 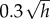 (*h* being the human density for the *i*th county) and s.d. 12. The concerned counties are Albany, Erie, Monroe, Nassau, Niagara, Rockland, Schenectady, Suffolk and Westchester. We plot in figure 4 the minimal (figure 4*a*) and maximal (figure 4*b*) initial populations stochastically generated by the procedure described earlier, and we report in table 3 the corresponding values.

**Table 3.** New York State: estimate of raccoon population. Minimal and maximal initial raccoon population for the counties included in the study.

county	min.	max.	county	min.	max.	county	min.	max.
1. Albany	10 595	24 885	20. Herkimer	29 039	60 441	39. St. Lawrence	56 409	1 21 406
2. Allegany	16 463	43 054	21. Jefferson	26 845	47 734	40. Saratoga	11 646	34 663
3. Broome	14 253	31 771	22. Lewis	25 472	51 688	41. Schenectady	4171	11 343
4. Cattaraugus	19 482	51 551	23. Livingston	11 322	25 528	42. Schoharie	10 948	25 644
5. Cayuga	11 567	28 961	24. Madison	11 431	26 676	43. Schuyler	6519	13 585
6. Chautauqua	15 383	40 411	25. Monroe	14 077	30 749	44. Seneca	5295	14 056
7. Chemung	7043	19 532	26. Montgomery	5163	15 227	45. Steuben	26 508	52 381
8. Chenango	17 840	33 385	27. Nassau	8600	16 983	46. Suffolk	21 985	46 092
9. Clinton	19 015	44 168	28. Niagara	11 861	23 499	47. Sullivan	18 035	38 966
10. Columbia	11 193	23 194	29. Oneida	16 844	49 377	48. Tioga	8029	19 435
11. Cortland	7285	22 220	30. Onondaga	14 216	36 543	49. Tompkins	9703	18 054
12. Delaware	22 355	61 437	31. Ontario	13 526	25 470	50. Ulster	20 425	46 220
13. Dutchess	15 692	33 017	32. Orange	14 761	41 116	51. Warren	14 664	35 641
14. Erie	24 051	56 638	33. Orleans	7136	14 544	52. Washington	14 131	34 818
15. Essex	31 369	66 867	34. Oswego	13 904	39 270	53. Wayne	11 001	26 300
16. Fulton	6647	22 492	35. Otsego	17 512	40 891	54. Westchester	14 462	25 374
17. Genesee	7491	19 424	36. Putnam	5270	10 650	55. Wyoming	10 081	24 834
18. Greene	10 976	26 937	37. Rensselaer	11 556	26 893	56. Yates	5669	14 404
19. Hamilton	31 505	78 448	38. Rockland	4850	9914

### 2.8. Model simulation and risk identification

**Figure 5.**
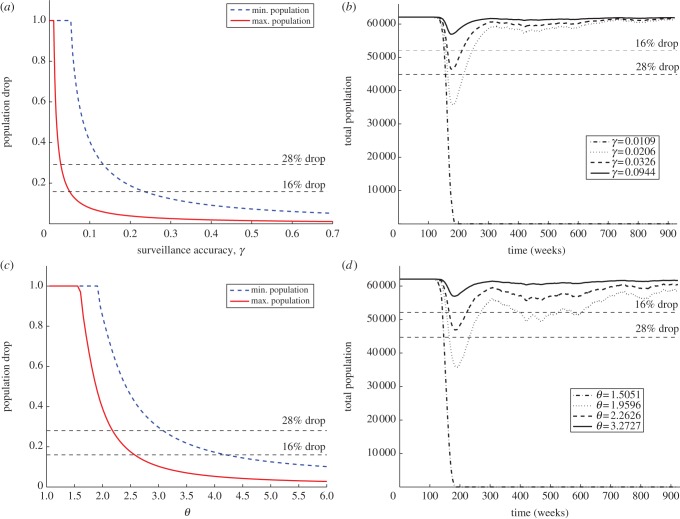
Albany county. (*a*,*b*) Constant surveillance, (*c*,*d*) dynamic surveillance. (*a*) Population drops in Albany county as a function of the surveillance accuracy *γ* for the estimated minimal and maximal population. (*b*) Temporal dynamics of the total population for different surveillance accuracies, given a maximal level as initial condition. (*c*) Population drops in Albany county as a function of the surveillance efficacy *θ* for the estimated minimal and maximal population. (*d*) Temporal dynamics of the total population for different surveillance efficacies, given a maximal level as initial condition. In (*a*–*d*), the horizontal dashed lines identify the range of population drop expected for raccoon rabies.

We ran simulations of the aggregated system with extrapolation from the data for all 56 counties with the 50 values of the initial population described earlier. The reports' behaviour along time seems to suggest the presence of an epidemic in almost all counties featuring positive reports, with the exception of Clinton, Hamilton, Suffolk and Warren, where the scarcity of reports does not allow us to draw evidence. The results for these counties have thus to be considered with care. We assumed that at the beginning of the epizootic the host population is entirely susceptible and at equilibrium, and that an epidemic has actually taken place in the counties included in the study. As a consequence, a drop in the population occurred that was compatible with the epizootic of the disease. We tested both the static and the dynamic model approaches, by running the SEI model with sampled values for *γ* and *θ*. Different values for *γ* and *θ* produce different temporal dynamics for the total population and different population drops (figure 5*b*,*d*). We sampled values of *γ* between 0 and 1, and values of *θ* between 1 and 7. For all 56 counties, we identify for all values of the initial population, the ranges of the parameters that produce population drops between 16% and 28% during the first outbreak.

Knowing the initial population, we can then assess the level of risk for each county (labelled by *i* = 1, … , 56) for the constant surveillance model. We choose the risk index 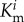, obtained algebraically from the midpoint of the compatibility interval for *γ*. If the compatible values of *γ* for the *i*th county lie in the interval 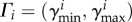, the corresponding risk index is given by 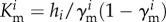, where *h_i_* is the human population of the county, and 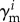 is the midpoint of the interval *Γ_i_*. The procedure clearly depends on the epidemic under study. In order to eliminate such dependence, we normalize the risk index to a scale from 1 to 10. Hence, we introduce for the *i*th county a surveillance risk *ρ*_*i*_, which is defined as the natural logarithm of 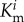 weighted by its maximum over all counties. The corresponding surveillance risk for th *i*th county is then given by
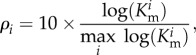
where a small value of *ρ*_*i*_ indicates a high level of surveillance in the county, whereas a large value of *ρ*_*i*_ entails a significant risk of an epidemic going undetected in the area.

In a similar manner, we can assess the surveillance efficacy for the dynamic surveillance model. In this case, we consider as indicator for the surveillance efficacy in the *i*th county, the value of *θ*_*i*_ corresponding to the midpoint of the interval associated with the initial population. A large value of *θ*_*i*_ indicates a high level of surveillance in the area, whereas a small value of *θ*_*i*_ highlights a significant risk that an epidemic will go undetected in the region.

Finally, the values of *ρ*_*i*_ and *θ*_*i*_ can be plotted on a geographical map to get a comprehensive view of the global risk across the state.

## 3. Results

Detailed results are shown for Albany County. This county has a very high count of reports, probably associated with the presence of the rabies diagnostic laboratory of the Wadsworth Center (New York State Health Department). We would like to observe that the presence of this large facility might induce bias in the estimated surveillance risk for the neighbouring counties. However, the observed disease dynamics are not different from what was observed in the majority of other counties. Figure 5*a*,*c* shows, respectively, for constant and dynamic surveillance, the curves obtained connecting the values of the parameters (*γ* and *θ*) paired with the associated population drop. The dashed blue line corresponds to the lower bound for the initial raccoon population, whereas the red line corresponds to the upper bound. The intersections of the two curves with the horizontal lines at 16% and 28% drop locate the intervals, where the compatibility constraints are satisfied. For static surveillance, we have 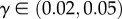 in the case we believe that the raccoon population is on the higher end of the estimate, and 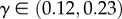 for the lower end. As we can see the lack of overlap between the compatibility intervals associated with the minimal and maximal initial population implies that optimal surveillance levels can be potentially very different. The importance of an accurate estimate of the initial raccoon population is crucial. A similar argument can be drawn for *θ* in the dynamic surveillance model, as shown in figure 5*c*.

Figure 5*b*,*d* shows different time series for the total raccoon population associated with different surveillance scenarios. We believe that the outbreak that occurred in Albany County was typical and we expect disease dynamics consistent with the values of *R*_0_ in the literature. For a population of roughly 60 000 raccoons, we can observe the drop caused by the outbreak, some damped oscillations and a slow recovery to the endemic equilibrium carrying capacity.

**Figure 6.**
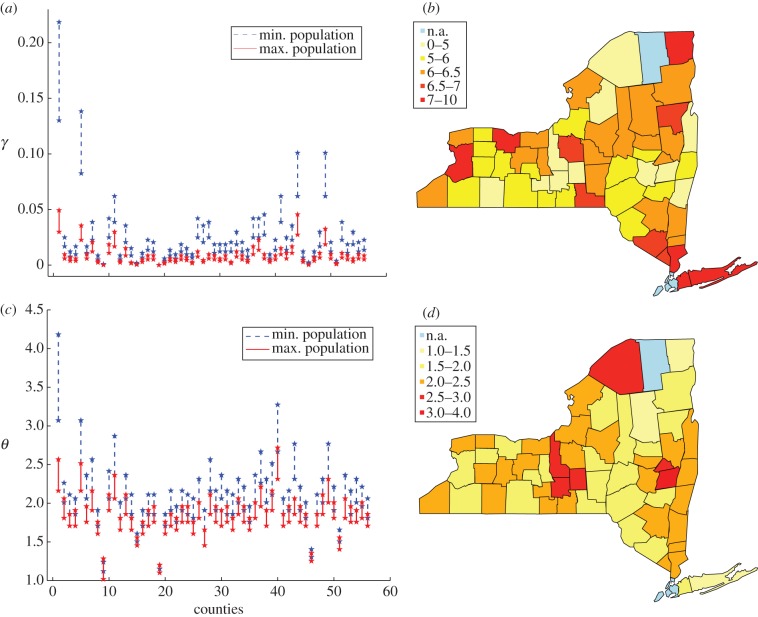
(*a*,*c*) Compatibility intervals for minimal and maximal initial raccoon population for the 56 New York counties with reported positive cases. Counties are numbered in alphabetical order matching table 1. (*a*) Constant surveillance, (*c*) dynamic surveillance. (*b*,*d*) Maps of surveillance risk and efficacy for the 56 New York counties with reported positive cases. (*b*) Surveillance risk map associated with constant surveillance. Small values indicate an high level of surveillance in the region, whereas large values entail a significant risk of an epidemic going undetected in the area. (*d*) Surveillance efficacy map associated with dynamic surveillance. Large values indicate a high level of surveillance in the area, whereas small values highlight a significant risk that an epidemics can go undetected in the region.

In figure 6*a*,*c*, we have comprehensive plots for *γ* and *θ* for the estimated intervals for all the 56 counties alphabetically ordered. The same level of surveillance can produce completely different interpretation of the disease dynamics: for instance, a value *γ* = 0.07 is associated with an outbreak so violent that it leads to extinction if the initial population is the minimal one, and at the same time with a complete absence of outbreak in the case where the initial population is the maximal one. Such a feature is shared by almost all the counties when a constant level of surveillance is assumed (figure 6*a*), with the exception of Clinton and Suffolk. In the case of dynamic surveillance, in contrast, only 12 counties do not feature an overlap between the intervals of *θ* corresponding to minimal and maximal initial population (figure 6*c*). Moreover, among those 12, only two feature a significant gap comparable with the length of the smaller interval (Albany and Schenectady).

Since the actual raccoon population is not known with absolute certainty, we choose to geographically map (figure 6*b*,*d*) the values of *ρ*_*i*_ and *θ*_*i*_ corresponding to the maximal estimated initial population. This is a conservative choice, justified by the consideration that the higher the population, the higher the risk (and relative consequences in terms of public health) of an undetected epidemic.

Finally, a somewhat expected duality between the intrinsic surveillance risk *ρ* associated with the constant extrapolation and the surveillance efficacy *θ* associated with the dynamic extrapolation is apparent, and can be assessed directly from the risk and efficacy mappings: areas with low surveillance risk display higher levels of surveillance efficacy.

## 4. Discussion

Surveillance is a key element in detecting, monitoring and studying infectious disease outbreaks over time and space. In this paper, we present some methodological aspects that can be used to evaluate the impact of localized surveillance for infectious diseases and help devising public health strategies. Intervention is based on information and the aim of this paper is to provide some of the information to decision-makers. As an illustration to the methodology, we showed an example based on a real dataset, consisting of positive and negative reported cases of rabid raccoons in the state of New York over a period spanning from 1990 to 2007.

We introduce two methods, both based on the idea of combining process-driven models with an observational approach, to take advantage of the features of both. The first method is based on a simple, constant reporting/detection rate, intended to model a constant level of surveillance over time. Considering that surveillance levels usually change because of news effects and public health concerns over possible outbreaks [18], we relax this assumption in our second model, where we formulate a reporting/detection rate that changes over time and depends on the total number of reports (positive and negative) and the estimated host population. Provided that such an estimate is accurate at any given time, it is possible to track disease dynamics through a model for disease spread [12]. With each of the two methods, we are able to identify locations where surveillance levels are critical and can potentially leave an outbreak unidentified.

The first method identifies surveillance risk, whereas the second one identifies a surveillance efficacy. An expected negative correlation between risk and efficacy emerged (−0.5652384). Besides being intuitive, such correlation is actually a sign that the two approaches are consistent, and either one can be used to identify areas at greater risk to which resources should be allocated in priority. The dynamic surveillance method (which assesses surveillance efficacy) provides results that are less sensitive to the initial population size. This aspect is very promising in view of extending the approach presented here to human diseases, where accurate accounts of the total population, with high resolution in space and more stable self reporting rates are available.

Two significant assumptions underlay our analyses. The first pertains to possible scenarios for the initial population size (before the first cases were recorded), and the second is that an epidemic actually occurred in each county where there was a positive reported case. We note that the first assumption is less limiting in the instance of human diseases. Since our study focuses on raccoon rabies, an *a priori* knowledge about the epidemiology of the disease is well known and established [29]. This is not a limiting aspect as long as the methodology is applied to extant diseases, but could prove problematic when applied to a newly emerging pathogen for which the epidemiology is not yet available. In this case, the method should be adapted by introducing some stochasticity in the key model parameters such as the transmission rate and the latency period.

Our work has the potential to be extended at both the methodological and applied level. For instance, the raccoon rabies surveillance analysis can potentially benefit from the inclusion of information regarding vaccinations programmes. Oral Rabies Vaccination (ORV) was initiated during the collection of our data (J. E. Childs 2000, personal communication) and may have affected, for instance, Essex and Clinton counties as suggested by a slight decline in the number of reports in those counties after ORV establishment. Unfortunately, we do not know if these modest declines are due to ORV or simply the decline is cases as the epizootic moved through the county. Very little is known about the rate of transition of individuals from the susceptible to the immune class through artificial immunization and we cannot, at this point, include such dynamics in our modelling. Investigating the efficacy of ORV programmes and verifying their eventual impact on disease dynamics might help better understanding of targeted surveillance, although it is unclear whether the conclusions we reached in our work will be sensitive to this extension.

Although uncertainty in outbreak size is taken into account by estimating system trajectories for different levels of *R*_0_ [21] and of initial host population [23], the model can be further generalized by including randomness in some of the parameters. A current work in progress involves estimation of parameters in a full Bayesian hierarchical setting. Combining the information from previous studies (prior elicitation) with the evidence arising from observational data (likelihood), we are able to produce estimates and uncertainty assessment for all the model parameters. This form of modelling bears directly on our understanding of the underlying disease process. Nonetheless, however the results can be incorporated also into the surveillance setting.

In future work, one could also estimate optimal levels of surveillance, by maximizing an utility function that depends on the social or environmental benefits of detecting an epidemic and on a penalty term with the costs associated with implementing surveillance policies. Furthermore, writing a stochastic model, possibly with the introduction of a spatial dynamics not considered in the present work [14,32–34], will allow us to actually estimate parameters and optimal surveillance levels in a likelihood framework. Finally, we also envision applications to other types of diseases where accurate estimates for the host population are available (for instance, some infectious diseases in humans).
